# YQWY Decoction Improves Myocardial Remodeling via Activating the IL-10/Stat3 Signaling Pathway

**DOI:** 10.1155/2020/7532892

**Published:** 2020-12-14

**Authors:** He Li, Zhi-Jun Gong, Yun He, Jing-Jing Huang, Yu-Ning Jiang, Ya-Yang Liu, Yao Zhu, Wei-Min Jiang

**Affiliations:** ^1^Department of Cardiology, The Affiliated Hospital of Nanjing University of Chinese Medicine, Jiangsu Province Hospital of Traditional Chinese Medicine, Nanjing, China; ^2^Department of Cardiology, Huai'an Hospital of Chinese Medicine, Huai'an, China; ^3^Department of Cardiology, The Affiliated Huai'an Hospital of Xuzhou Medical University, Huai'an, China; ^4^Institute of Hypertension, Jiangsu Province Hospital of Traditional Chinese Medicine, The Affiliated Hospital of Nanjing University of Chinese Medicine, Nanjing, Jiangsu, China

## Abstract

Heart failure (HF) has been known as a global health problem, and cardiac remodeling plays an essential role in the development of HF. We hypothesized that YQWY decoction might exert a cardioprotective effect against myocardium inflammation, fibrosis, and apoptosis via activating the interleukin-10 (IL-10)/Stat3 signaling pathway. To test this hypothesis, the HF model in rats was established by pressure overload through the minimally invasive transverse aortic constriction (MTAC). Echocardiography was performed to assess the left ventricular function of rats. Myocardial fibrosis in rats was observed by Masson and Picrosirius red staining, and the degree of myocardial apoptosis was detected via TUNEL staining. In addition, expression levels of IL-10, tumor necrosis factor-*α* (TNF-*α*), Stat3 (P-Stat3), P65 (P-P65), CD68, collagen I, TGF-*β*, CTGF, Bax, Bcl-2, cleaved caspase-3, and PARP in rat serum and myocardium samples were examined by ELISA, western blot, and immunohistochemistry, respectively. YQWY decoction treatment significantly improved left ventricular function in HF rats, especially in those of the high-dose group (LVEF%: 51.29 ± 5.876 vs. 66.02 ± 1.264, *P* < 0.01;, LVFS%: 27.75 ± 3.757 vs. 37.76 ± 1.137, *P* < 0.01). Furthermore, YQWY decoction markedly inhibited MTAC-induced myocardial fibrosis as evidenced by downregulated collagen I, TGF-*β*, and CTGF in myocardium and alleviated apoptosis (downregulated caspase-3 and PARP and increased Bcl-2/Bax ratio in cardiomyocytes). In addition, YQWY decoction decreased the level of the proinflammatory cytokine TNF-*α* in both circulating blood and myocardium and attenuated infiltration of inflammatory cells in heart tissue from HF rats. Most importantly, YQWY decoction suppressed MTAC-induced NF-*κ*B activation and phosphorylated Stat3 by upregulating IL-10 in rat heart tissues. Our study showed that YQWY decoction could attenuate MTAC-induced myocardial inflammation, fibrosis, apoptosis, and reverse the impairment of cardiac function in rats by activating the IL-10/Stat3 signaling pathway and improving myocardium remodeling. Our findings suggested a therapeutic potential of YQWY decoction in HF.

## 1. Introduction

Heart failure (HF) affects more than 2% of the general population, which is a major public health concern that costs a high economic burden on the health system [1]. Despite years of decline in the incidence of HF, it remains severe conditions with substantial morbidity, mortality, and frequent hospitalizations.

Cardiac remodeling has been considered as a key factor in the progress of HF [2]. Many clinical studies have demonstrated that reversal of cardiac remodeling can alleviate the progress of HF [3, 4]. It is well known that increased cardiac hypertrophy, apoptosis, fibrosis, and altered metabolism are characteristic features of cardiac remodeling [3]. Several important signaling pathways, such as cell death signaling, calcium handling, myocardial energy metabolism, oxidative stress, and neurohormonal activation are involved in the pathophysiology of myocardial dysfunction and remodeling [5]. Although some agents targeting neurohumoral activation process, such as renin-angiotensin-aldosterone system (RAAS) inhibitors and *β*-adrenergic receptor blockaders, have been considered as first-line treatments that are beneficial to the prognosis of HF [6, 7], morbidity and mortality of HF remain high in clinical practice. Therefore, exploring the underlying molecular mechanism of cardiac remodeling is of great significance to develop new therapeutic strategies for HF.

It is worth mentioning that interleukin-10 (IL-10) is an effective potential target for the treatment of cardiac remodeling [8]. IL-10 presents the most potential anti-inflammatory and anti-immune activities, which is mainly produced by T-helper cells and monocytes/macrophages [9]. In addition to the regulation of Th1 cytokines, IL-10 also attenuates the synthesis of inflammatory cytokines from monocytes/macrophages, including IL-1, tumor necrosis factor-*α* (TNF-*α*), IL-8, and IL-12 [10]. Recently, growing evidence has indicated the relationship between IL-10 and cardiovascular diseases (CVD), particularly atherosclerosis, and IL-10 is believed to be a potential predictor for CVD risk [11]. It is further observed that treatment with IL-10 contributes to protecting the left ventricular function, attenuates cardiac inflammation, inhibits fibrosis, and improves ventricular remodeling in myocardial infarction or pressure overload mouse model [8, 12]. The anti-inflammatory function of IL-10 is mainly regulated via activating the signal transducer and activator of transcription 3 (Stat3) [10, 13]. Further experimental studies showed that an inhibition of IL-10/Stat3 in myocardial tissues recruits inflammation cells, damages the function of the heart, and enhances mortality after myocardial infarction (MI) in mice [14]. Therefore, IL-10/Stat3 is expected to be a novel therapeutic target for heart disease treatments.

A large number of investigations have suggested the combined application of herbal and chemical drugs in preventing and treating diseases nowadays. Yi Qi Wen Yang (YQWY) decoction has been commonly used in the treatment of HF in the Jiangsu Province Hospital of Traditional Chinese Medicine (TCM). We have previously reported that the treatment of YQWY decoction suppresses the cardiomyocyte hypertrophy as well as reverses the damaged heart function [15]. In the present study, potential effects of YQWY on cardiac remodeling, myocardial inflammation, fibrosis, and apoptosis of HF rats were mainly explored, as well as the underlying mechanism.

## 2. Materials and Methods

### 2.1. Animals and Surgeries

Male Wistar rats (240∼300g, 8 weeks old) were purchased from the Vital River Laboratory Animal Technology Co. Ltd. All rats were raised in the SPF-level, Drug Evaluation Center of Nanjing University of TCM, with the temperature of 20–25°C and the relative humidity of 50%–70%. Autoclaved and sterilized feed and hygienic drinking water were given. After one-week adaptive feeding, the rat HF model was established by MTAC. In brief, rats were anesthetized with a mixture of 2% isoflurane and 0.5∼1.0 L/min 100% O_2_ in an induction chamber. After fixing in a hypsokinesis position on a heating pad, partial thoracotomy of the second rib was performed. A 27-gauge blunt needle was placed parallel to the transverse aorta, which was removed after two knots using 6–0 silk suture were tied. Rats in the sham group underwent the same surgery without constriction.

### 2.2. Preparation of YQWY Decoction

The prescription of YQWY was provided by the Cardiology Department of the Affiliated Hospital of Nanjing University of Chinese Medicine. It is composed of 60 g raw radix of *Astragalus membranaceus* (Fisch.) Bge. (Astragali Radix), 15 g radix and rhizome of *Rhodiola crenulata* (Hook.f. et Thomes.) H Ohba. (Radix Rhodiolae), 10 g radix of *Aconitum carmichaeli* Debx. (Aconiti Lateralis Radix Preparata), 15 g sclerotium of *Polyporus umbellatus* (Pers.) Fries (Polyporus), 15 g seeds of *Descurainia Sophia* (L.) Webb. Ex Prantl. (Descurainia semen), 10 g rhizome of *Curcuma phaeocaulis* Val. (Curcumae Rhizoma), 12 g radix of *Paeonia lactiflora* Pall. (Paeoniae Radix Alba), and 6 g rhizome of *Zingiber officinale* Rosc. (Zingiberis Rhizoma Recens). All of the herbal medicine was purchased from the Affiliated Hospital of Nanjing University of Chinese Medicine and morphologically identified by Dr. Fu-Qiong Zhou at Nanjing Chinese Hospital of Nanjing University of Chinese Medicine. Furthermore, YQWY was concentrated into liquid for convenient use. The combined filtrate was concentrated under reduced pressure and evaporated to dryness. Finally, 27.84% (w/w) YQWY water extract was yielded. The concentration of the decoction in the low-dose group was calculated to be 12.8 g raw materials/kg/d (3.6 g YQWY water extract/kg/d), which was equivalent to the dose of the adult. A concentration of 18 g YQWY water extract/kg/d was applied in the high-dose group, which was 5 times the equivalent dose of the adult.

### 2.3. Preparation of Myocardial Tissues and Blood Samples

After sacrifice, rat hearts were taken out and washed in normal saline. The LV tissue was rapidly separated from the atria, which was fixed in 4% paraformaldehyde for 24 h and then embedded in paraffin. Blood samples were taken from the abdominal aorta. After centrifugation for 10 min at 3000 × g at 4°C, the serum was extracted and frozen at −80°C until use.

### 2.4. Cardiac Imaging

After the successful establishment of the HF rat model, the rat was anesthetized by isoflurane and placed in the supine position. The high-frequency color ultrasound system Vevo 2100 with a resolution of 40 *μ*m, up to 300, was provided by the ultrasound laboratory of the Jiangsu Provincial Medical Animal Experimental Base, which was used for obtaining the 2D image of the sternal LV short axis. Doppler and M-mode echocardiography was performed perpendicular to the interventricular septum and the posterior wall of the LV at the level of the papillary muscle, which was used to measure left ventricular ejection fraction (LVEF) and left ventricular shortening rate (LVFS). The average of three cardiac cycles was taken continuously.

### 2.5. Enzyme-Linked Immunosorbent Assay (ELISA)

Serum and myocardium samples were collected from rats. Relative concentrations of IL-10 and TNF-*α* were detected in them using a commercial ELISA kit (RayBiotech, Norcross, GA, USA).

### 2.6. Masson's Trichrome Staining

Paraffin-embedded sections were dewaxed and diluted in distilled water. They were stained in Weigert iron-staining dyeing solution for 5–8 minutes and differentiated 1% hydrochloric acid alcohol for a few seconds and Lichun red acid magenta dye solution for 3–4 minutes. Between each interval, tissue sections were washed for several minutes. Later, sections were induced in 1% phosphomolybdic acid solution and differentiated for about 5 minutes, dried, and washed directly with aniline blue dye solution for 5 minutes. After incubating in 1% glacial acetic acid for 1 minute, they were rapidly washed and dehydrated in 95% alcohol and anhydrous ethanol. We observed the sections which were permeated with xylene, dried, and sealed with neutral glue under the microscope.

### 2.7. Sirius Red Staining

Paraffin-embedded sections were dewaxed, dyed in Celestin blue B for 5–10 minutes, and washed in distilled water three times. Subsequently, they were induced in prepared Sirius red staining solution for 15–30 minutes, followed by differentiation, dehydration, penetration, and sealing. Sirius red-stained sections were observed under a microscope.

### 2.8. Immunohistochemistry (IHC) Analysis

Positive expressions of CD68, collagen I, TGF-*β*, and CTGF in heart tissues were evaluated by IHC. Isolated heart tissues were placed in 4% paraformaldehyde and fixed for 12 h, followed by dehydration and embedding with paraffin. These tissue-contained wax blocks were then prepared into sections with 4 *μ*m thickness. After antigen retrieval in citrate, the sections were incubated with protein blocking buffer (pH 7.5) (ab126587, Abcam) for an hour at room temperature (RT). The samples were then incubated with specific primary antibodies at 4°C overnight, followed by incubation with HRP-labeled anti-rabbit antibody for 60 minutes at 37°C. Antibodies adopted are shown in Table S1. Later, the slices were intervened with DAB substrate kit (ab64238, Abcam) for 10 minutes, and the nuclei were stained with hematoxylin for another 4 minutes. Five visual fields were randomly selected from each sample, and the images were recorded using the Olympus BX43 microscope and DP73 camera (Olympus, Tokyo, Japan). Antibodies used in the experiment are listed in the supplementary material (Table S1).

### 2.9. Western Blot Analysis

The protein of heart tissues was extracted *via* RIPA (Beyotime, Jiangsu, China) containing 1% PSMF, and the concentration was determined with BCA kits (Beyotime, Jiangsu, China). The sample concentration was then adjusted to 60 *μ*g using the 5 × SDS-PAGE sample loading buffer, and the diluted sample was heated to 100°C for 5 minutes. The denatured proteins were separated using 10% SDS-PAGE and transferred to the polyvinylidene difluoride membranes. Protein blocking buffer (pH 7.5) was used to block the polyvinylidene difluoride membranes at RT for 1 h. Subsequently, the polyvinylidene difluoride membranes were intervened with specific primary antibodies at 4°C for 12 h. After washing with TBST three times, they were incubated with HRP-conjugated secondary antibodies (1 : 10,000) for 1 h at 37°C. Protein blots were visualized using a VersaDoc imaging system (Bio-Rad, USA). GAPDH was used as a loading control. Antibodies used in the experiment are listed in the supplementary material (Table S1).

### 2.10. Terminal Deoxynucleotide Transferase-Mediated dUTP Nick End Labeling (TUNEL)

Tissue sections were induced in permeabilization solution and blocking buffer and washed in PBS for at least three times. Then, they were incubated with TUNEL solution (Roche Applied Science, Indianapolis, IN, USA) for 60 minutes at 37°C. Immediately, sections were incubated with 0.1 *μ*g/mL 4,6-diamidino-2-phenyl-indole (DAPI) for nuclei staining. The fluorescent staining was conducted by using an Olympus BX43 microscope and captured using the DP73 camera.

### 2.11. Statistical Analysis

All data were expressed as mean ± standard deviation (SD) and performed using GraphPad Prism 7 (GraphPad Software Inc., San Diego, CA, USA). One-way ANOVA was used to determine statistical difference among three or more groups, and $\underline{P}$ value equal to or <0.05 was considered to be statistically significant.

## 3. Results

### 3.1. YQWY Protected Cardiac Function of HF Rats

**Figure 1:**
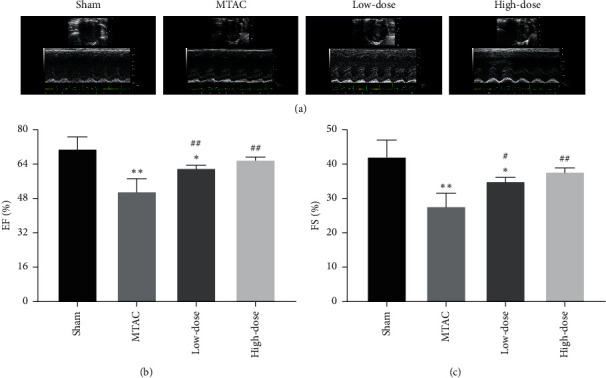
YQWY protected cardiac function of HF rats: (a) representative echocardiography images of LV; (b) LVEF in each group; (c) FS in each group. Values were expressed as mean ± SD, *n* = 5. ^*∗*^*P* < 0.05 and ^*∗∗*^*P* < 0.01 vs. sham group. ^#^*P* < 0.05 and ^##^*P* < 0.01 vs. MTAC group.

To determine the effects of YQWY on protecting the cardiac function of HF rats, cardiac echocardiography was conducted after 16-week interventions. Doppler and M-mode echocardiography revealed shorter LVFS and LVEF in the MTAC group than those of the sham group (*P* < 0.05). However, YQWY-treated rats showed increased LVEF and LVFS than those of the MTAC group, indicating the improved LV function (Figures 1(a)–1(c)).

### 3.2. YQWY Alleviated MTAC-Induced Cardiac Fibrosis

**Figure 2:**
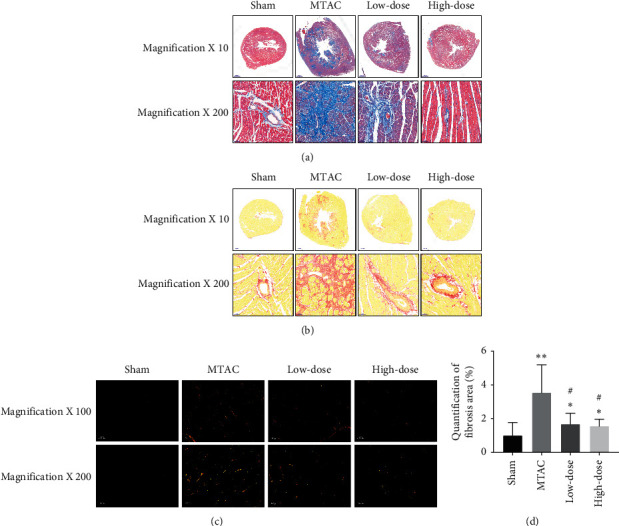
YQWY alleviated MTAC-induced cardiac fibrosis: (a) representative images of Masson's staining; (b, c) representative images of Sirius red staining under the light microscopy (b) and polarized light microscopy (c); (d) quantification of fibrosis. Values were expressed as mean ± SD, *n* = 5. ^*∗*^*P* < 0.05 and ^*∗∗*^*P* < 0.01 vs. sham group. ^#^*P* < 0.05 and ^##^*P* < 0.01 vs. MTAC group.

Cardiac fibrosis is a crucial event in the pathological process of ventricular remodeling. Therefore, we assessed fibrotic changes in HF rats with YQWY treatment. Masson's staining was employed to detect the collagen deposition in LV sections. The results indicated that the myocardial fibrosis degree in the TAC group was remarkably higher than that in the sham group (Figure 2(a)). Meanwhile, representative images of Sirius red staining showed that YQWY alleviated MTAC-induced cardiac fibrosis (Figures 2(b)–2(d)). Both the low-dose and high-dose groups presented attenuated cardiac fibrosis than the MTAC group (*P* < 0.05), which was more pronounced in the high-dose group.

### 3.3. YQWY Suppressed the Cardiac Fibrotic Signaling in HF Rats

**Figure 3:**
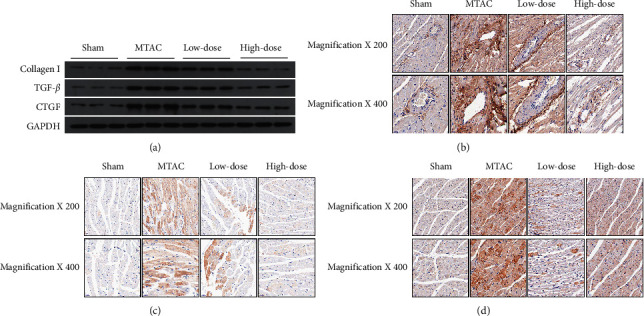
YQWY suppressed the cardiac fibrotic signaling in HF rats. (a) Representative western blot bands of collagen I, TGF-*β*, and CTGF; (b–d) IHC analysis of collagen I (b), TGF-*β* (c), and CTGF (d) in rat heart sections.

To further investigate the effect of YQWY on protecting myocardial fibrosis, fibrosis genes (collagen I, TGF-*β*, and CTGF) were examined. Protein levels of collagen I, TGF-*β*, and CTGF were markedly upregulated in the MTAC group than those of the sham group, which were downregulated by YQWY treatment (Figure 3(a)). It is suggested that YQWY was able to alleviate fibrosis post-HF. Identically, IHC data also showed decreased positive expressions of collagen I (Figure 3(b)), TGF-*β* (Figure 3(c)), and CTGF (Figure 3(d)) in heart tissues of YQWY-treated rats. Altogether, our results demonstrated an in vivo cardiac protective role of YQWY in HF rats.

### 3.4. YQWY Inhibited MTAC-Induced Cardiomyocyte Apoptosis

**Figure 4:**
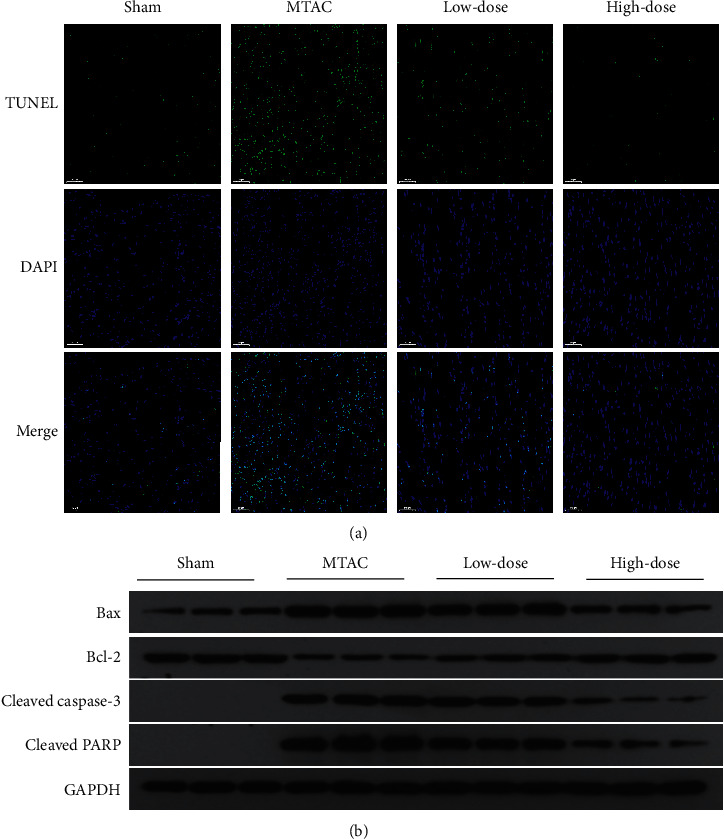
YQWY inhibited MTAC-induced cardiomyocyte apoptosis. (a) Representative images of TUNEL staining; (b) representative western blot bands of Bax, Bcl-2, cleaved caspase-3, and cleaved PARP.

TUNEL staining results showed that YQWY could inhibit cardiomyocyte apoptosis in heart sections of HF rats (Figure 4(a)). Subsequently, we detected protein levels of apoptotic genes (Bax, Bcl-2, cleaved caspase-3, and cleaved PARP) by western blot. Cleaved caspase-3 and PARP were markedly upregulated after MTAC operation, which were suppressed by YQWY treatment. Bcl-2 was remarkably downregulated in the MTAC group, while Bax was upregulated. Their expression trends were remarkably reversed in the high-dose group and low-dose group (Figure 4(b)). It is concluded that YQWY treatment was able to inhibit cardiomyocyte apoptosis in HF rats.

### 3.5. YQWY Inhibited Inflammatory Response in HF Rats

**Figure 5:**
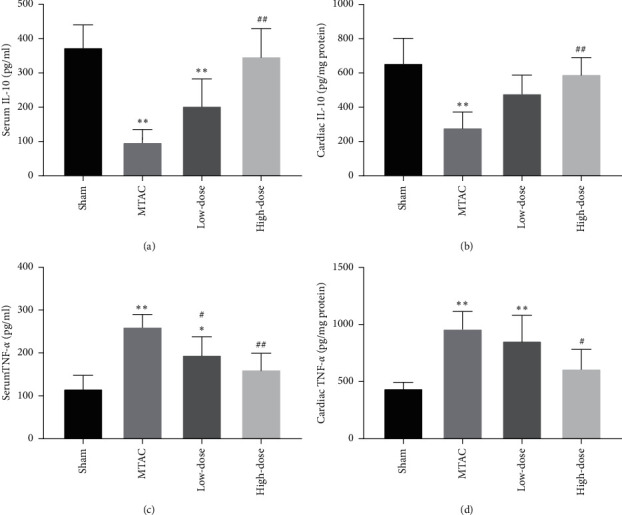
YQWY inhibited inflammatory response in HF rats. (a, b) YQWY treatment increased IL-10 levels in the serum (a) and myocardium (b) of HF rats; (c, d) YQWY decreased TNF-*α* levels in the serum (c) and myocardium (d) of HF rats. Values were expressed as mean ± SD, *n* = 5. ^*∗*^*P* < 0.05 and ^*∗∗*^*P* < 0.01 vs. sham group. ^#^*P* < 0.05 and ^##^*P* < 0.01 vs. MTAC group.

Relative levels of inflammatory factors in serum and myocardium samples were detected. IL-10 levels in both serum and myocardium samples of the MTAC group were reduced than those of the sham group, whilst those of TNF-*α* were elevated (*P* < 0.05). Notably, decreased levels of IL-10 and increased levels of TNF-*α* were dramatically reversed by YQWY treatment (*P* < 0.05) (Figures 5(a)–5(d)). Taken together, YQWY was capable of inhibiting inflammatory response following the development of HF through upregulating IL-10 and downregulating TNF-*α*.

### 3.6. YQWY Protected HF Rats through Activating the IL-10/Stat3 Signaling and Inactivating the NF-*κ*B-P65 Signaling

**Figure 6:**
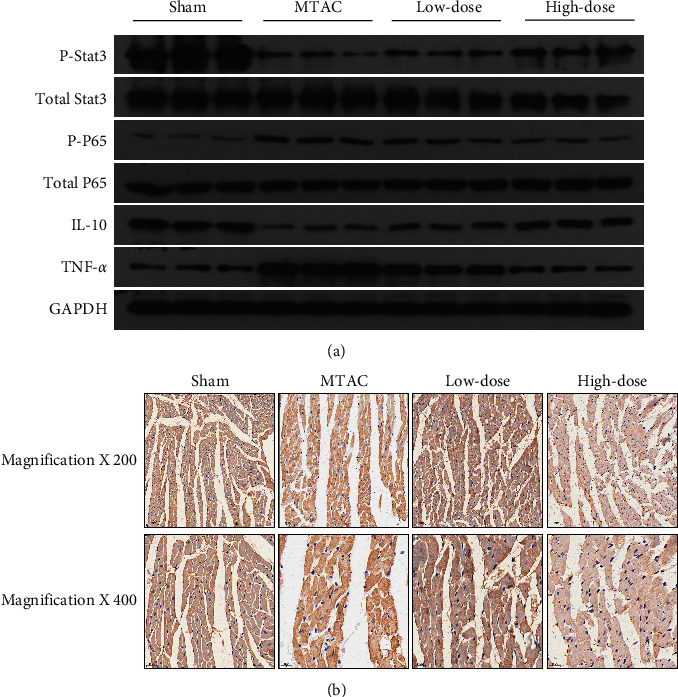
YQWY protected HF rats through activating the IL-10/Stat3 signaling and inactivating the NF-*κ*B-P65 signaling. (a) Representative western blot bands of IL-10, P-Stat3, Stat3, P-P65, P65, and TNF-*α*; (b) IHC analysis of CD68 in heart sections.

IL-10 has been shown to track the translocation of P65 and participate in the regulation of NF-*κ*B activation via interacting with the Stat3 signaling [16]. Here, protein levels of IL-10 and P-Stat3 were significantly downregulated in the MTAC group, while P-P65 and its downstream gene TNF-*α* were upregulated (Figure 6(a)). Expression changes of IL-10, P-Stat3, P-P65, and TNF-*α* were markedly reversed by YQWY treatment (Figure 6(a)). According to IHC results, we further confirmed that YQWY acted as an inhibitor of CD68, a well-known marker of inflammatory cell infiltration (Figure 6(b)). Therefore, YQWY was confirmed to activate the IL-10/Stat3 signaling and inactivate the NF-*κ*B-P65 signaling, thus downregulating the downstream cytokine TNF-*α*.

## 4. Discussion

In the present study, we focused on the cardioprotective effects of YQWY decoction, a Chinese herb compound, on pressure overload-induced HF rats by MTAC. Our finding demonstrated that YQWY decoction markedly improved LV function in HF rats as LVFS and LVEF were enhanced. Importantly, YQWY decoction also significantly reduced pressure overload-induced left ventricular fibrosis and cardiomyocyte apoptosis. Furthermore, YQWY decoction inhibited inflammatory response and attenuated cardiac infiltration of inflammatory cells in HF rats. IL-10, an anti-inflammatory cytokine, plays a cardioprotective role in ventricular remodeling. Interestingly, our results showed upregulated IL-10 and downregulated NF-*κ*B in HF rats treated by YQWY decoction, which may be explained by the phosphorylation of Stat3. Taken together, our study indicated that YQWY decoction reduced inflammation, fibrosis, and apoptosis in myocardium and improved LV function in HF rats through activating the IL-10/Stat3 signaling pathway.

Despite significant advances in Western medication for the treatment of HF, novel strategies that can effectively and safely fight against heart diseases are urgently required. TCM is commonly used as a complementary therapeutic approach of HF in China for a long time [17]. Recently, several clinical trials and animal experiments have demonstrated the beneficial effects of Chinese herbs on the management of HF [18, 19]. However, their fundamental mechanisms in the treatment of HF remain to be exactly elucidated.

YQWY decoction has been used for the treatment of HF, which is composed of eight herbs as follows: Astragali Radix, Rhodiolae Radix, Aconiti Lateralis Radix Preparata, *Polyporus,* Descurainia Semen, Curcumae Rhizoma, Paeoniae Radix Alba, and Zingiberis Rhizoma Recens. In the present study, an in vivo pressure overload-induced HF rat was established by performing MTAC. Rat LV function was evaluated by echocardiography. Our results showed that LV function was markedly impaired in HF rats and YQWY decoction improved rat heart function as evidenced by increased LVEF and LVFS. Consistently, astragaloside IV, the major active component of Astragali Radix, has been reported to improve cardiac function and downregulate BNP and ANP in TAC-induced pressure overload models [20]. Calycosin, another bioactive component of Astragali Radix, is able to reverse impaired LV systolic function in myocardial infarction rats [21]. Rhodiolae Radix is beneficial to increase cardiac output in streptozotocin-induced diabetic rats with HF [22]. Components of YQWY decoction are complicated and its pharmacological role against HF needs to be testified in the future.

The pathogenesis of HF is complex and cardiac remodeling has been recognized as the critical determinant of HF progression. Therefore, targeting therapy for pathological cardiac remodeling is now thought to be the key event in the management of HF. Myocardial fibrosis and apoptosis are early indicators of HF. Myocardial fibrosis is characterized by elevated amounts of ECM proteins, which increases ventricular stiffness and impairs cardiac relaxation and contractility [23]. Apoptosis is of significance in the development of ventricular remodeling, which markedly decreases myocardial mass [24]. Here, we firstly assessed fibrosis in HF rats. Compared to the sham group, cardiac fibrosis was more pronounced in HF rats, which was attenuated by YQWY treatment via downregulating collagen I, TGF-*β*, and CTGF in the heart. Furthermore, the antiapoptotic role of YQWY decoction in HF rats was assessed. Abundant TUNEL-positive cells were observed in the heart of HF rats, which were remarkably reduced by YQWY treatment. As expected, activities of caspase-3 and PARP were reduced by YQWY treatment, while the Bcl-2/Bax ratio was enhanced. It is concluded that YQWY treatment markedly alleviated MTAC-induced cardiac fibrosis and apoptosis.

Notably, accumulated evidences have demonstrated that persistent inflammation is a key contributor to the initiation and progression of cardiac remodeling, which plays a central role in the development of HF [25]. Changes of cardiac inflammation are mediated by infiltration of neutrophils, monocytes/macrophages, and lymphocytes in myocardium. Enhanced proinflammatory cytokines secreted from these immune cells may contribute to the pathogenesis of myocardium dysfunction and the syndrome of HF [26, 27]. Multiple clinical reports have revealed that patients with HF have elevated levels of inflammatory cytokines, such as TNF-*α*, IL-1*β*, MCP-1, and IL-8 in circulation and myocardial tissues [28, 29]. Increased circulating level of TNF-*α* appears to be associated with the deterioration of left ventricular function [30]. It is well established that anticytokine treatments, including TNF-*α*, IL-1*β*, and IL-8, are capable of improving cardiac function and outcomes in animal experiments [31–33]. Recently, IL-10, a kind of anti-inflammatory cytokine, has been demonstrated to protect ventricular function and cardiac remodeling. IL-10 knockout mice exhibit significantly decreased left ventricular function and increased fibrosis and cell death in myocardium compared to wild-type mice induced with isoproterenol [8]. Following TGF-*β*-induced cardiac fibrosis, expression levels of collagen I and *α*-smooth muscle actin (*α*-SMA) in myocardium tissues are remarkably upregulated, which are partially reversed by IL-10 intervention [34]. IL-10 is able to decrease caspase-3 activity and increase Bcl-2/Bax ratio, which alleviates TNF-*α*-induced apoptosis in cardiomyocytes [35]. Our study showed that expression levels of IL-10 in serum and myocardium tissues of HF rats were significantly lower than controls, which were elevated after YQWY decoction treatment. We also found that YQWY decoction reduced the cardiac level of TNF-*α* and inhibited cardiac infiltration of inflammatory cells in HF rats. Therefore, we considered that YQWY decoction protected cardiac function of HF rats by maintaining the balance between the pro- and anti-inflammatory cytokines.

NF-*κ*B activation is a vital event in the cardiac pathological remodeling and progression of HF [36, 37]. P65, one of the major NF-*κ*B subunits, is a key regulator of myocardia hypertrophy that promotes adverse ventricular remodeling [38, 39]. Inactivation of P65 by overexpressing phosphorylation-resistant IkB-*α* mutant is conductive to protect ventricular function and prevent apoptosis and inflammation in HF mice [40]. IL-10 is capable of suppressing multiple proinflammatory responses. Recent studies have demonstrated that IL-10 blocks NF-*κ*B activity in cardiac myocytes in a Stat3-dependent manner [8]. Here, decreased activation of Stat3 and upregulated P-P65 were observed in heart tissues of HF rats, which were restored by YQWY decoction, suggesting that the cardioprotective effect of YQWY decoction was associated with the activated IL-10/Stat3 pathway.

## 5. Conclusion

YQWY decoction could attenuate myocardial inflammation, fibrosis, and apoptosis and repair cardiac function in MTAC-induced HF rats through activating the IL-10/Stat3 signaling pathway and improving myocardium remodeling. Great efforts are required in the future on validating the efficacy and mechanism of TCM in the treatment of HF.

## Data Availability

The datasets used and/or analyzed during the current study are available from the corresponding author upon reasonable request.

## Abbreviations

ANOVA: Analysis of variance
ACEIs: Angiotensin-converting enzyme inhibitors
CTGF: Connective tissue growth factor
ELISA: Enzyme-linked immunosorbent assay
GATA4: GATA binding protein 4
HF: Heart failure
IL-10: Interleukin-10
LVEF: Left ventricular ejection fraction
LV: Left ventricular
LVFS: Left ventricular shortening fraction
MOM: Mitochondrial outer membrane
MAPKs: Mitogen-activated protein kinases
NF-*κ*B: Nuclear factor *κ*B
PARP: Peak-to-average power ratio
P13K: Phosphoinositide 3-kinase
TUNEL: Terminal deoxynucleotidyl transferase-mediated dUTP nick end labeling
RT: Room temperature
Stat3: Signal transducer and activator of transcription
TGF-*β*: Transforming growth factor-beta
TTE: Transthoracic echocardiography
MTAC: Minimally invasive transverse aortic constriction
TNF: Tumor necrosis factor
VEGF: Vascular endothelial growth factor
VMC: Viral myocarditis
WB: Western blot.

## Supplementary Materials

Supplementary Materials Table S1: primary antibodies used in the IHC and WB experiments.
