# Supplementary material for: YQWY Decoction Improves Myocardial Remodeling via Activating the IL-10/Stat3 Signaling Pathway
Source: Evid Based Complement Alternat Med. 2020 Dec 14;2020:7532892. doi: 10.1155/2020/7532892 (PMC7787750; doi:10.1155/2020/7532892)
Supplement: Supplementary Materials — Table S1: primary antibodies used in the IHC and WB experiments. [file 7532892.f1.doc]

**Supplementary material**

**Table S1** Primary antibodies used in the IHC and WB experiments.

| Antibody | Company | Species | Catalog | Dilution | Application |
| --- | --- | --- | --- | --- | --- |
| IL-10 | Abcam | Rabbit | Ab9969 | 1:1000 | WB |
| P-Stat3 | Cell Signaling Technology | Rabbit | 9145 | 1:2000 | WB |
| Stat3 | Cell Signaling Technology | Rabbit | 12640S | 1:1000 | WB |
| P65 | Cell Signaling Technology | Rabbit | 8242 | 1:1000 | WB |
| P-P65 (Ser563) | Cell Signaling Technology | Rabbit | 3033 | 1:1000 | WB |
| TNF-α | SANTA CRUZ | Mouse | Sc-52746 | 1:200 | WB |
| CD68 | Abcam | Rabbit | Ab125212 | 1:500 | IHC |
| Collagen I | Abcam | Rabbit | Ab34710 | 1:2000 | WB |
|  |  |  |  | 1:200 | IHC |
| TGF-β | Abcam | Rabbit | Ab92486 | 1:2000 | WB |
|  |  |  |  | 1:200 | IHC |
| CTGF | Proteintech | Rabbit | 23936-1-AP | 1:500 | WB |
|  |  |  |  | 1:200 | IHC |
| Bax | Cell Signaling Technology | Rabbit | 14796S | 1:1000 | WB |
| Bcl-2 | Invitrogen | Mouse | 33-6100 | 1:1000 | WB |
| Cleaved Caspase-3 | Abcam | Rabbit | Ab2302 | 1:1000 | WB |
| Cleaved PARP | Abcam | Rabbit | Ab32561 | 1:1000 | WB |
| GAPDH | Abcam | Rabbit | Ab181602 | 1:10000 | WB |
